# Risk factors for difficulty of laparoscopic cholecystectomy in grade II acute cholecystitis according to the Tokyo guidelines 2013

**DOI:** 10.1186/s12893-017-0319-6

**Published:** 2017-11-28

**Authors:** Koetsu Inoue, Tatsuya Ueno, Daisuke Douchi, Kentaro Shima, Shinji Goto, Michinaga Takahashi, Takanori Morikawa, Takeshi Naitoh, Chikashi Shibata, Hiroo Naito

**Affiliations:** 1 Department of surgery, South Miyagi Medical Center, 38-1 Aza-nishi, Ogawara, Shibata-gun, Miyagi 989-1253 Japan; 2 Department of surgery, Tohoku University Graduate School of Medicine, 1-1, Seiryo-machi, Aoba-ku, Sendai 980-8574 Japan; 3 Division of Gastroenterological Surgery, Department of Surgery, Tohoku Medical and Pharmaceutical University Hospital, 1-12-1 Hukumuro Miyagino-ku, Sendai, Miyagi 983-8512 Japan

**Keywords:** Acute cholecystitis, Laparoscopic cholecystectomy, Tokyo guidelines 2013

## Abstract

**Background:**

The Tokyo Guidelines 2013 classifies acute cholecystitis (AC) into three grades and recommends appropriate therapy for each grade. For grade II AC, either early laparoscopic cholecystectomy (LC) or percutaneous transhepatic gallbladder drainage (PTGBD) should be performed. This study aimed to identify the risk factors for difficulty of LC for treating grade II AC.

**Methods:**

Totally, 122 patients who underwent LC for grade II AC were enrolled and divided into difficult LC (DLC) and nondifficult LC (NDLC) groups. The DLC group included patients who experienced one of the following conditions: conversion from LC to open cholecystectomy, operating time ≥ 180 min, or blood loss ≥300 ml. Preoperative characteristics and postoperative outcomes were analyzed.

**Results:**

In univariate analysis, risk factors included male sex, interval between symptom onset and admission, interval between symptom onset and LC, and anticoagulant therapy. The incidence of postoperative complications was higher in the DLC group than in the NDLC group (23.5% vs. 4.6%, *p* = 0.0016). According to receiver operating characteristic curves, the optimal cutoff value was calculated, and multivariate analysis showed that male sex [odds ratio (OR), 5.76; 95% confidence interval (CI), 1.979–19.51; *p* = 0.0009) and interval between symptom onset and LC of over 96 h (OR, 6.32; 95% CI, 2.126–20.15; *p* = 0.0009) were independent risk factors for difficulty of LC.

**Conclusions:**

In patients with grade II AC, LC was technically difficult when performed over 96 h after symptom onset. Moreover, male sex was a risk factor. Therefore, PTGBD should be considered in these patients.

**Electronic supplementary material:**

The online version of this article (10.1186/s12893-017-0319-6) contains supplementary material, which is available to authorized users.

## Background

Acute cholecystitis (AC) is commonly observed, and laparoscopic cholecystectomy (LC) has become the standard treatment for this disease [1, 2]. The advantages of LC over open cholecystectomy include shorter hospital stay, reduced postoperative pain, and less mortality and morbidity [3–5]. Recently, the superiority of early LC over delayed LC was demonstrated in a randomized controlled trial [6–11]; however, surgeons often encounter difficulties in performing LC due to their inability to correctly identify the anatomy of Calot’s triangle as a result of severe inflammation. Therefore, in patients with severe AC, the rate of complications, such as bile leakage, common bile duct injury, and bowel injury, is high after LC [12, 13], suggesting the importance of evaluation of inflammation severity.

**Fig. 1:**
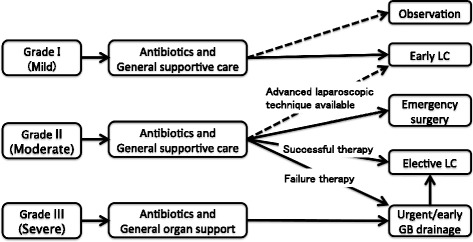
Treatment according to AC grade and response. LC: Laparoscopic cholecystectomy, GB: Gallbladder

The Tokyo Guidelines 2007 was issued as the first international guideline for the diagnosis and treatment of AC; recently, it was revised to the Tokyo Guidelines 2013 (TG13) [14, 15]. The TG13 suggests criteria for the classification of AC based on clinical symptoms and physical examination, blood test, and imaging findings. According to the TG13, AC is classified into three grades: grade I (mild), grade II (moderate), and grade III (severe) (Table 1). The TG13 also recommends appropriate therapy depending on the grade of AC (Fig. 1). Patients with grade I AC are candidates for undergoing immediate LC, those with grade II AC can undergo either early LC or percutaneous transhepatic gallbladder drainage (PTGBD), and those with grade III AC are strongly recommended to undergo immediate PTGBD. For patients with grade II AC, either early LC or PTGBD should be performed when antibiotic therapy is not effective. The TG13 recommends performing early LC in medical centers where skilled surgeons can operate. Therefore, it is important to consider risk factors for difficulty of LC in patients with grade II AC. Although some studies have reported risk factors for difficulty of LC with a particular focus on conversion from LC to open cholecystectomy, their evaluations were not based on the grading of AC according to the TG13 [16–22].

**Table 1:** Classification of acute cholecystitis by the Tokyo guidelines 2013

Grade	Definition	
I (Mild)	Acute cholecystitis that does not meet the criteria for Grade III or Grade II	
	Acute cholecystitis in a healthy patient with no organ dysfunction. Inflammatory changes in the gallbladder are mild, making cholecystectomy a safe and low-risk procedure.	
II (Moderate)	Grade II acute cholecystitis is associated with any one of the following conditions	
	1	Elevated white blood cell count (>18,000/mm^4^)
	2	Palpable tender mass in the right upper abdominal quadrant
	3	Duration of complaints > 72 h
	4	Marked local inflammation (gangrenous cholecystitis, pericholecystic abscess, hepatic abscess, biliary peritonitis, emphysematous cholecystitis)
III (Severe)	Grade III acute cholecystitis associated with dysfunction of any one of the following organs/systems	
	1	Cardiovascular dysfunction defined as hypotension requiring treatment with ≥ 5 μg/kg per min of dopamine or any dose of norepinephrine
	2	Neurologic dysfunction defined as decreased level of consciousness
	3	Respiratory dysfunction defined as a PaO2/FiO2 ratio of <300
	4	Renal dysfunction defined as oliguria or creatinine level of > 2.0 mg/dl
	5	Hepatic dysfunction defined as PT-INR of > 1.5
	6	Hematologic dysfunction defined as platelet count of <100,000/mm^3^

This study aimed to assess risk factors affecting the technical difficulty of LC in patients with grade II AC.

## Methods

**Fig. 2:**
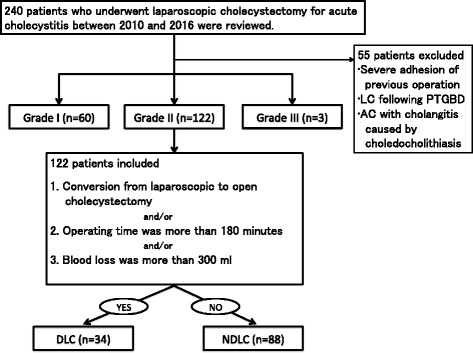
Study flow chart. AC: Acute cholecystitis. LC: Laparoscopic cholecystectomy. PTGBD: Percutaneous transhepatic gallbladder drainage. DLC: Difficult laparoscopic cholecystectomy. NDLC: Nondifficult laparoscopic cholecystectomy

The medical records of 240 patients who underwent LC for AC between 2010 and 2016 were retrospectively reviewed (Fig. 2). Totally, 55 patients who met one of the following conditions were excluded: tight adhesion due to a previous upper abdominal operation, LC following PTGBD, or AC with cholangitis caused by choledocholithiasis. At least one board-certified surgeon of the Japan Surgical Society participated in all operations.

As there was no quantitative evaluation for difficulty of cholecystectomy, we used objective and subjective criteria [16, 20]. Difficult LC was defined as the presence of one of the following conditions: conversion from laparoscopic cholecystectomy to open cholecystectomy, operating time ≥ 180 min, or blood loss ≥300 ml. The criteria for conversion was as follows: other organ injury, refractory bleeding, and difficulty identifying important structures inside Calot’s triangle. The decision for conversion was made by each surgeon. The remaining 122 patients with grade II AC were divided into two groups according to the aforementioned criteria: the difficult LC (DLC; *n* = 34) and nondifficult LC(NDLC; *n* = 88) groups. Preoperative characteristics and postoperative outcomes were further analyzed. This study protocol was reviewed and approved by the Ethics Committee of the South Miyagi Medical Center.

Data are shown as mean ± standard error of the mean. Categorical variables were analyzed by the chi-square test, whereas continuous data were analyzed by either the two-tailed Student’s *t*-test or Wilcoxon test, based on the results of the Shapiro–Wilk test. Multivariate analysis was performed with logistic regression analysis. Statistical significance was defined as *p* < 0.05. The optimal cutoff value was calculated using the receiver operating characteristic (ROC) curve and was defined as the number that indicated the highest sum of the sensitivity and specificity on the ROC curve. Statistical analysis was performed with JMP Pro 11 (SAS Institute, Cary, NC, USA). Detailed data of patient characteristics are shown in Additional file 1.

## Results

Among the enrolled patients, AC was classified as grade I in 60 patients (32.4%), as grade II in 122 (65.9%), and as grade III in 3 (1.7%). Among 122 patients with grade II AC, 33 were classified into the DLC group. There were 18, 15, and 16 patients whose operating time was >180 min, whose intraoperative blood loss was >300 ml, and whose procedure was changed to open cholecystectomy, respectively. Four patients fulfilled all three abovementioned criteria. The most common reason for conversion was the inadequate exposure of Calot’s triangle due to severe adhesion and inflammation. Additionally, accidental cystic duct injury in three patients contributed to conversion. In one patient, the cystic duct was injured during exposure of Calot’s triangle. In the other two patients, the dissection of necrotic and fragile cystic ducts using a linear stapler resulted in bile leakage from the stump of the cystic duct. No patient had gallbladder cancer.

Detailed patient characteristics are shown in Table 2. The ratio of male-to-female patients was significantly higher in the DLC group (82.4% vs. 58.0%; *p* = 0.011). Intervals between symptom onset and LC (126 ± 9.04 vs. 70.3 ± 5.62 h; *p* < 0.0001) and between symptom onset and admission (65.6 ± 7.07 vs. 32.9 ± 4.40 h; *p* = 0.0001) were significantly longer in the DLC group than in the NDLC group. The percentage of patients receiving anticoagulant therapy was higher in the DLC group than in the NDLC group (29.4% vs. 13.6%, *p* = 0.042). All other parameters did not differ between the groups.

**Table 2:** Comparison of patient clinical characteristics

Variable	DLC group	NDLC group	*P* value
	(*n* = 34)	(*n* = 88)	
Age, years	68.8 ± 2.42	66.7 ± 1.50	0.455
Male, *n* (%)	28 (82.4)	51 (58.0)	0.011
BMI	24.5 ± 0.672	25.2 ± 0.418	0.389
Interval between symptom onset and operation, hours	126 ± 9.04	70.3 ± 5.62	< 0.0001
Interval between symptom onset and admission, hours	65.6 ± 7.07	32.9 ± 4.40	0.0001
Fever on admission, *n* (%)			0.715
No	24 (70.6)	65 (73.9)	
Yes	10 (29.4)	23 (26.1)	
Abdominal pain on admission, *n* (%)			0.409
No	1 (2.9)	6 (6.8)	
Yes	33 (97.1)	82 (93.2)	
ASA score, *n* (%)			0.949
<3	11 (32.4)	29 (32.9)	
≥3	23 (67.6)	59 (67.1)	
Performance status, *n* (%)			0.695
<3	32 (94.1)	81 (92.1)	
≥3	2 (5.9)	7 (7.9)	
Anticoagulant therapy, *n* (%)	10 (29.4)	12 (13.6)	0.042
Hypertension, *n* (%)	12 (35.3)	39 (44.3)	0.365
Diabetes mellitus, *n* (%)	9 (26.5)	20 (22.7)	0.663

Continuous variables are presented as mean ± SEM

*BMI* Body mass index, *ASA* American Society of Anesthesiologists

Table 3 shows laboratory data and imaging findings. Laboratory data, including inflammation-associated parameters, such as white blood cell count and C-reactive protein levels, did not differ between the groups. No difference was observed between the groups in terms of the thickness of the gallbladder wall and existence of a gallbladder stone and/or sludge (Table 3).

**Table 3:** Comparison of laboratory data and imaging findings

Variable	DLC group	NDLC group	*P* value
	(n = 34)	(*n* = 89)	
Laboratory data			
White blood cells, 10^3^/μL	14.6 ± 0.893	14.3 ± 0.555	0.780
Hemoglobin, g/dL	13.9 ± 0.326	14.0 ± 0.203	0.791
Platelets, 10^3^/μL	198 ± 9.42	201 ± 5.86	0.788
CRP, mg/dL	13.0 ± 1.60	10.1 ± 0.993	0.120
Total bilirubin, mg/dL	1.76 ± 0.195	1.45 ± 0.121	0.19
AST, IU/L	42.8 ± 21.0	79.9 ± 13.0	0.136
ALT, IU/L	41.9 ± 14.5	70.0 ± 9.0	0.102
ALP, IU/L	310 ± 39.0	321 ± 24.2	0.816
γ-GTP, IU/L	113 ± 28.3	114 ± 17.6	0.984
Radiographic and ultrasonographic findings			
GB wall thickness, mm	6.53 ± 0.435	5.85 ± 0.271	0.183
GB stone and/or sludge, n (%)	34 (100)	84 (95.5)	0.206

Continuous variables are presented as mean ± SEM

*CRP* C-reactive protein, *AST* Aspartate aminotransferase, *ALT* Alanine aminotransferase, *ALP* Alkaline phosphatase, *γ-GTP* Gamma-glutamyl transpeptidase, *GB* Gallbladder

Perioperative characteristics are shown in Table 4. Operating time and blood loss were greater and the rate of subtotal cholecystectomy and/or mucoclasis was significantly higher in the DLC group than in the NDLC group. The percentage of patients with necrosis and/or abscess did not differ between the groups.

**Table 4:** Comparison of perioperative characteristics

Variable	DLC group	NDLC group	*P* value
	(*n* = 33)	(n = 89)	
Operating time, min	178 ± 6.32	122 ± 3.93	<0.0001
Operating time ≥ 180 min, n (%)	18 (52.9)	0 (0)	
Blood loss, ml	354 ± 31.4	45.1 ± 19.1	<0.0001
Blood loss ≥300 ml, n (%)	15 (44.1)	0 (0)	
Open conversion, n (%)	16 (47.1)	0 (0)	<0.0001
Subtotal cholecystectomy and/or mucoclasis, n (%)	20 (58.8)	19 (21.6)	<0.0001
Necrosis and/or abscess, n (%)	28 (82.4)	65 (73.9)	0.323

Continuous variables are presented as mean ± SEM

Perioperative outcomes are shown in Table 5. The DLC group had a significantly higher incidence of postoperative complications than the NDLC group (23.5% vs. 4.6%; *p* = 0.0016). In particular, bile leakage and surgical site infection developed more frequently in the DLC group than in the NDLC group (11.8% vs. 1.1%; *p* = 0.0079 for bile leakage and 5.9% vs. 0%; *p* = 0.0218 for surgical site infection). The percentage of patients with Clavien–Dindo classification ≥II was significantly higher in the DLC group than in the NDLC group (17.7% vs. 4.5%; *p* = 0.018); however, the number of patients with Clavien–Dindo classification ≥IIIa did not differ between the groups. The duration of postoperative hospital stay was longer in the DLC group than in the NDLC group (9.26 ± 0.60 days vs. 5.89 ± 0.37 days; *p* < 0.0001).

**Table 5:** Comparison of postoperative outcomes

Variable	DLC group	NDLC group	*P* value
	(*n* = 34)	(*n* = 88)	
Postoperative complications, *n* (%)			0.0016
Yes	8 (23.5)	4 (4.6)	
No	26 (76.5)	84 (95.4)	
Bile leakage, *n* (%)	4 (11.8)	1 (1.1)	0.0079
Pulmonary complication, *n* (%)	2 (5.9)	1 (1.1)	0.129
Surgical site infection, *n* (%)	2 (5.9)	0 (0)	0.0218
Intraabdominal abscess, *n* (%)	0 (0)	1 (1.1)	0.533
Colitis, *n* (%)	0 (0)	1 (1.1)	0.533
Clavien–Dindo classification			
≥ II, *n* (%)	6 (17.7)	4 (4.6)	0.018
≥ IIIa, *n* (%)	3 (8.8)	2 (2.3)	0.102
Postoperative hospital stay, days	9.26 ± 0.60	5.89 ± 0.374	< 0.0001

Continuous variables are presented as mean ± SEM

Table 6 shows the results of multivariate analysis for risk factors for difficult of LC. ROC curve analysis yielded a value of 96 h as the optimal cutoff interval between symptom onset and LC, whereas a value of 72 h was the optimal cutoff interval between symptom onset and admission. With these cutoff values, multivariate analysis was performed. Interval between the symptom onset and LC over 96 h [odds ratio (OR), 6.32; 95% confidence interval (CI), 2.126–20.15; *p* = 0.0009) and male sex (OR, 5.76; 95% CI, 1.979–19.51, *p* = 0.0009) were independent risk factors for difficulty of LC.

**Table 6:** Results of multivariate analysis

Variable	*P* value	Odds ratio	95% confidence interval
Male, *n* (%)	0.0009	5.76	1.979–19.51
Interval between symptom onset and operation ≥96 h, n (%)	0.0009	6.32	2.126–20.15
Interval between symptom onset and admission ≥72 h, *n* (%)	0.246	2.03	0.6124–6.870
Anticoagulant therapy, *n* (%)	0.258	1.96	0.6054–6.238

## Discussion

Recently, early LC has become a standard treatment for AC [23, 24]; however, surgeons often experience difficulty during LC for AC as a result of severe local inflammation, which can increase the rate of postoperative complications, such as bile leakage, common bile duct injury, and bowel injury [12]. The TG13 has indicated severity criteria for AC and has recommended its appropriate management [15]. For grade II AC, however, surgeons can select either early LC or PTGBD. The TG13 recommends early LC by a well-experienced surgeon; therefore, it is important to consider risk factors for difficulty of LC while determining the appropriate treatment. Although some studies have reported risk factors for difficulty, they did not evaluate them according to each grade of AC as indicated by the TG13. Therefore, risk factors for difficulty of LC in grade II AC remain unclear.

In this retrospective study, we assessed risk factors for difficulty of LC in 122 patients with grade II AC. The conversion rate was 13.1%. Because previous studies had a conversion rate ranging from 12.9% to 24% [16, 19], the conversion rate in our study was acceptable. Although some investigators defined difficult LC according to the conversion rate, the decision for conversion depends on the skill of the surgeon and is very subjective. Thus, the conversion rate differs among surgeons. Operating time and blood loss are well-accepted parameters that affect postoperative complications [20]. Therefore, operating time and blood loss were also included in the definition. In the DLC group, the incidences of postoperative complications and postoperative hospital stay were significantly greater than those in the NDLC group. Moreover, the rate of subtotal cholecystectomy and/or mucoclasis was significantly higher in the DLC group. Accordingly, we considered that the definition for technical difficulty of LC was appropriate.

Male sex, interval between symptom onset and LC, interval between symptom onset and admission, and anticoagulant therapy were considered as candidates for risk factors in univariate analysis; however, multivariate analysis revealed that male sex and interval between symptom onset and LC were independent risk factors. In some patients receiving anticoagulant therapy, the timing of LC was delayed so that patients could recover from the effect of drugs. Hence, there was a confounding bias between the risk factors anticoagulation therapy and interval between symptom onset and LC. Alternatively, we evaluated the effect of antibiotics on the difficulty of LC in patients with grade II AC. Initially, we hypothesized that the early administration of antibiotics makes it easy to complete LC; however, we could not prove this hypothesis in our study. Therefore, an early decision should be made for LC regardless of antibiotic therapy.

Regarding postoperative complications, bile leakage occurred significantly more often in the DLC group than in the NDLC group. Fortunately, there was no major bile duct injury requiring bile duct reconstruction in our study; however, this result indicated that minor bile duct injury during operation occurred more often in the DLC group due to tight adhesion around the gallbladder neck. There were more patients with Clavien–Dindo classification ≥II in the DLC group than in the NDLC group, and they had a prolonged postoperative hospital stay. Meanwhile, the numbers of patients with Clavien–Dindo classification ≥IIIa did not differ between the groups.

Interval between symptom onset and LC has been reported as a risk factor for conversion, and the critical point ranged from 48 h to 96 h [16, 18, 25]. In our study, the cutoff value was 96 h, which was similar to previous data. The edematous phase, which is the phase before proceeding to tight adhesion, predominates in this period [25]; therefore, the TG13 recommends early LC.

Ambe et al. [26] suggested that male sex was a risk factor for severe gallbladder inflammation in AC and for complications in patients with AC undergoing LC [22]. Yajima et al. [27] also reported that male sex was a risk factor for conversion from laparoscopic cholecystectomy to open cholecystectomy. Male sex was also an independent risk factor for difficult LC in our study. Ambe et al. discussed that male patients gave poor attention to their health compared to female patients, and consequently, their visit to the hospital was delayed. Therefore, male patients have a long interval between symptom onset and LC. In our study, however, no significant difference was observed in the interval between symptom onset and LC. There may be other reasons for this finding. Although previous studies also reported that older age, high white blood cell count, and high C-reactive protein levels are essential risk factors [16, 17, 19, 20], there was no difference in these variables in our study.

Our study had two limitations. First, our study was retrospective and had a relatively small sample size. Second, the decision for conversion was made by each surgeon according to subjective criteria. These two limitations can be confounding factors for perioperative outcomes, including operating time, blood loss, and conversion rate.

## Conclusions

LC was technically difficult when performed later than 96 h after symptom onset in patients with grade II AC. Moreover, male sex was a risk factor. Therefore, PTGBD should be considered in these patients.

## Additional file

Additional file 1: Detailed data of patient characteristics. 1: yes, 0: no (XLSX 61 kb)

## Abbreviations

AC: Acute cholecystitis
CI: Confidence interval
DLC: Difficult laparoscopic cholecystectomy
LC: Laparoscopic cholecystectomy
NDLC: Nondifficult laparoscopic cholecystectomy
OR: Odds ratio
PTGBD: Percutaneous transhepatic gallbladder drainage
ROC: Receiver operating characteristic
TG13: Tokyo Guidelines 2013
